# Analysis of cell-free DNA concentration, fragmentation patterns and *TP53* gene expression in mammary tumor-bearing dogs: A pilot study

**DOI:** 10.3389/fvets.2023.1157878

**Published:** 2023-03-29

**Authors:** Silvia Guil-Luna, Raquel Sánchez-Céspedes, Aurora Rivas Crespo, María Dolores Fernández, José Andrés Fernández Sarmiento, Antonio Rodríguez-Ariza, Yolanda Millán

**Affiliations:** ^1^Grupo Nuevas Terapias en cáncer, Instituto Maimónides de Investigación Biomédica de Córdoba, Córdoba, Spain; ^2^Centro de Investigación Biomédica en Red de Cáncer (CIBERONC), Madrid, Spain; ^3^Departamento de Anatomía y Anatomía Patológica Comparadas, Facultad de Veterinaria de Córdoba, Universidad de Córdoba, Córdoba, Spain; ^4^Departamento de Medicina y Cirugía Animal, Facultad de Veterinaria, Universidad de Córdoba, Córdoba, Spain

**Keywords:** canine mammary tumors, liquid biopsy, p53 mutation detection, cell-free DNA, tumor biomarkers

## Abstract

**Introduction:**

Liquid biopsy based on the analysis of circulating cell-free DNA (cfDNA), as well as on detection of point mutations by digital droplet PCR (ddPCR), has revolutionized the research in oncology. In recent years, this technique has been pioneering in veterinary medicine since it is a minimally invasive approach with very promising results for characterization of tumors.

**Methods:**

The aim of this study was, firstly, to analyze the concentration and the fragmentation pattern of cfDNA of dogs with mammary tumors (*n* = 36) and healthy dogs (*n* = 5) and its correlation with clinicopathological data. Secondly, analysis of *TP53* gene expression and the point mutation in the codon 245 were performed in cfDNA and in tumor tissues to assess their potential as plasma biomarkers.

**Results and discussion:**

Our results highlighted that those dogs with worse clinicopathological characteristics (simple or undifferentiated carcinomas, higher histological grade and presence of peritumoral inflammation) shown higher cfDNA concentration and higher concentrations of short-fragments (<190 bp) than healthy dogs. In addition, although no detection of the point mutation in codon 245 of *TP53* gene could be detected neither in plasma nor tumor tissue, an increased *TP53* expression was detected in animals with tumors bearing malignant characteristics. Finally, a high concordance with *TP53* gene expression in plasma and tumor tissue and cfDNA concentration was also found. The results derived from this work confirm the valuable potential of cfDNA and its fragments, as well as the analysis of *TP53* expression in plasma as useful liquid biomarkers for clinical application in veterinary oncology.

## 1. Introduction

In the era of precision cancer medicine, circulating cell-free DNA (cfDNA) liquid biopsy has received enormous attention due to its huge potential in human oncology (1). In contrast to tissue sampling, liquid biopsy is emerging as a minimally invasive tool which is not limited by sampling frequency or tumor accessibility (1). Thus, it offers the potential for providing rapid results for the diagnosis, prognosis and progression of cancer patients. cfDNA is an extracellular short double-stranded DNA released into the bloodstream or any body fluid, as a result of release mechanisms including apoptosis, necrosis, senescence, NETosis and active secretion (2). In cancer patients, circulating tumor DNA (ctDNA) is the principal component of cfDNA since it is shed by tumor cells and contains the same genetic and epigenetic alterations (mutations, copy number alterations, chromosomal rearrangements, hyper and hypomethylation) present in the primary tumor (3, 4). Several studies have confirmed that high levels of cfDNA in the peripheral blood are associated with tumor progression and poor prognosis in cancer patients (4–6). In addition, not only tumor progression and prognosis, but also its proliferation rate, have been positively correlated to the amount of cfDNA (7–9). Thus, the analysis of cfDNA have drastically revolutionized the field of oncology in the last years offering ease in tumor sampling, early diagnosis, disease staging and for the monitoring of disease progression (4, 10).

In veterinary medicine, cfDNA analysis in canine tumors are still very limited with a huge need for more fundamental research on its origin and kinetics. As in humans, higher plasma cfDNA concentrations have been positively correlated with the severity of several canine malignancies including canine mammary tumors (CMTs) (10–13). Additionally, the presence of genomic alterations in cfDNA in several canine tumors has opened new opportunities for characterizing tumor mutational landscapes with many applications in comparative oncology (14–18).

Recent studies has revealed that cfDNA from tumor cells is generally more fragmented [160–190 pair of basis (bp)] than non-tumoral cfDNA (19, 20). Thus, the analysis of cfDNA fragmentation patterns is currently a tumor biomarker for the prediction of tumor prognosis and malignancy in human oncology (4–6, 19, 20). In veterinary medicine, until now, just one study has analyzed cfDNA fragments in CMTs by qPCR of two amplicons to assess its potential to differentiate benign and malignant lesions (12).

At present, there is an increased focus on the analysis in cfDNA of cancer-related genomic alterations, such as mutations in *KRAS, TP53, PIK3CA*, or *p16* genes, by using new highly sensitive technologies such as digital droplet PCR (ddPCR), BEAMing (beads, emulsion, amplification and magnetics) or next-generation sequencing (NGS) approaches (21). In this sense, *TP53* overexpression and point mutations are one of the most common genetic abnormalities detected in up to 20% of CMTs comparable to that of human breast carcinoma (22, 23). As well as tumor mutational analysis of ctDNA shows numerous clinical applications in human medicine, the detection of point mutations in cfDNA in veterinary oncology is currently receiving increasing interest but with limited studies so far. Thus, overexpression of the protein p53 and *TP53* genetic alterations observed in human and CMTs, has been associated with cancer cell proliferation, invasion, metastasis and drug resistance (24–26). The high frequency of TP53 mutations found in tumor cells makes it a highly promising target for veterinary oncology so its detection by liquid biopsy can be used as a tumor biomarker with great potential in veterinary oncology.

Based on the above, in this study, firstly, we determined the cfDNA concentration and the fragmentation pattern in plasma of dogs with CMTs and healthy dogs were analyzed and, secondly, this information was correlated it with clinicopathological data and the molecular classification of the tumors. Moreover, we analyzed the *TP53* gene expression and the point mutation in the codon 245 of *TP53* gene in cfDNA and its respective tumor tissue to assess its correlation and possible potential as a liquid biomarker in CMTs.

## 2. Materials and methods

### 2.1. Case selection and clinicopathological data

A cohort of 36 female dogs undergoing resection of mammary tumors (malignant and benign tumors) by radical or partial mastectomy at the Veterinary Hospital of the University of Córdoba was included in the study. In addition, five healthy female dogs without any signs of pathological disease were selected as control group. Clinicopathological data of each dog, as well as the informed consent from each owner, was also prospectively collected for the study. The study was approved by the Ethical Committee of University of Córdoba in accordance with the Code of Ethics of the World Medical Association (Declaration of Helsinki).

### 2.2. Blood and tumor sample collection

Blood and fresh tissue tumor samples from bitches were collected during the surgery. Additionally, around 6–10 ml of blood was taken from each dog in ethylenediaminetetraacetic acid (EDTA) tubes. Immediately after blood collection, plasma was separate by centrifugation at 1,600 × g during 10 min at room temperature (RT) followed by centrifugation at 6,000 × g during 10 min at RT to remove any possible cell debris. Plasma samples were then aliquoted, transferred to cryotubes and stored at −80°C before DNA isolation. Fresh tumor samples were sliced and fixed in 10% buffered formaldehyde for 24 hours and processed for paraffin embedding for histological and immunohistochemical (IHC) analyses.

### 2.3. Histological and IHC analysis of tumor samples

The histological diagnosis was confirmed on hematoxylin and eosin (H&E)-stained full-sections by two independent pathologists (YMR and RSC) according to Zapulli et al. (27). In addition, assessment of tumor inflammatory cell infiltration and presence of necrosis within the tumor (yes/no) was performed on H&E-stained full sections (28).

For IHC analysis, four-μm-thick sections were obtained and placed on Vectabond-coated slides (Sigma Diagnosis, St Louis, Missouri) and immunostaining was performed using mouse/rabbit anti-human monoclonal antibodies previously standardized in canine samples and listed in Supplementary Table S1. The slides were deparaffined, rehydrated in a graded series of ethanol and incubated with 3% hydrogen peroxide in methanol for 30 min. Heat-induced antigen retrieval was performed in a water bath at 96°C with 0.01 M citrate buffer (pH 6) for 15 min. Then, sections were covered with 10% normal goat serum in phosphate buffer saline for 30 min before incubation with the primary antibody for 18 h at 4°C. Afterwards, the avidin-biotin-peroxidase complex method was applied as recommended by the manufacturer (Vector Laboratories, Burlingame, California) followed by 3,3′diaminiobenzidine (DAB) staining (Agilent, Santa Clara, CA, USA). Sections were then counterstained with hematoxylin, dehydrated and mounted. As positive control tissues, human breast carcinoma tissue was used for HER2, canine uterus tissue was used for progesterone receptor (PR) and estrogen receptor (ER) antibodies, canine epithelial mammary tissue was used for CK5 and CK14 and canine duodenum tissue was used for Ki67. The normal mammary gland found in the tissue sections under study was used as an internal positive control in every assay. As negative controls, the primary antibodies were replaced immunoglobulin fraction of serum from non-immunized rabbits (Agilent, Santa Clara, CA, USA) for anti-HER2 and anti-ER, mouse IgG2 (Agilent, Burlingame, CA, USA) for anti-PR, and mouse IgG1 (Agilent, Santa Clara, CA, USA) for anti-CK5, anti-CK14 and Ki67 antibodies.

### 2.4. Molecular classification of canine mammary carcinomas

For molecular classification of canine mammary carcinomas (CMC), the classification previously described by Sánchez-Céspedes et al. (28) was used. Immunostaining of anti-HER2, -PR, -ER, -CK5, -CK14, and Ki67 were semi-quantitatively analyzed based on the intensity and percentage of positive tumor cells. To evaluate HER2 expression, the 2018 ASCO/CAP guidelines were followed; 10 selected fields with the strongest protein expression were evaluated, and those cases with 3^+^ [complete membrane staining >10 % of positive luminal epithelial (LE) tumor cells] were considered HER2 positive (29).

PR and ER labeling were assessed using the Allred score in 10 randomly selected fields (30), following a semi-quantitative system that accounts for the staining intensity (scored on a scale of 0–3: 0 = none; 1 = weak; 2 = intermediate, and 3 = strong) and the proportion of positive cells (scored on a scale of 0–5: 0 = none; 1 = < 1%; 2 = 1%−10%; 3 = 10%−33%; 4 = 33%−66 %, and 5 = 66%−100%), regardless of their myoepithelial (ME) or epithelial nature. The intensity and proportion of the immunolabelling against each antibody, ER and PR, were evaluated together yielding a total score of 0 to 8. A score of ≥3 was considered positive (30).

For the IHC evaluation of CK5 and CK14 expression, both epithelial and myoepithelial cells (when presented in complex and mixed tumors), 10 randomly selected fields were evaluated. Cases with ≥10 % of positive tumor cells, without considering the residual/pre-existing myoepithelial cells, and with strong cytoplasmic staining were considered to be positive (28). Ki67 expression was used to determine the proliferation index (PI). To do so, images were captured from four fields at high power field magnification (40 × microscope objective) with high number of Ki67-positive cells. The number of Ki67-positive and Ki67-negative epithelial cells was assessed by image analysis using ImageJ software (https://imagej.net/Fiji). The PI was expressed as the percentage of positively-labeled cells. A minimum of 1,000 tumor cells were counted per case (28).

**Figure 1:**
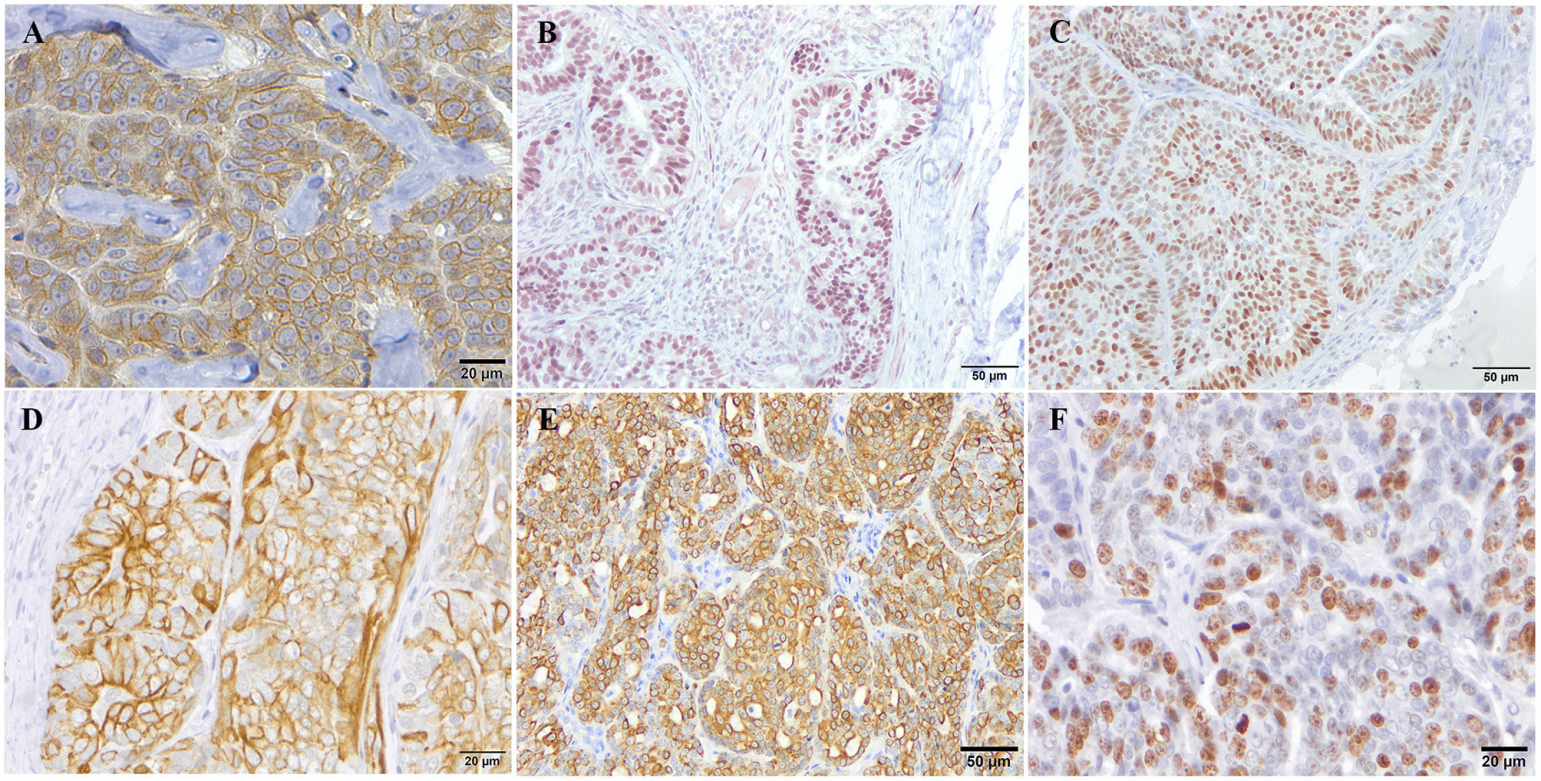
Immunohistochemical expression pattern of the different markers used for the molecular classification of canine mammary tumors. Representative immunohistochemical staining for HER-2 **(A)**, PR **(B)**, ER **(C)**, CK5 **(D)**, CK14 **(E)**, and Ki67 **(F)** antibodies.

The 26 CMCs were grouped into molecular subtypes as follows: luminal A (ER^+^ and/or PR^+^, Ki67^low^, and CK5 ^±^ or CK14 ^±^); luminal B (ER^+^ and/or PR^+^, Ki67^high^, and CK5 ^±^ or CK14 ^±^); HER2-overexpression (HER2^+^, ER–, PR–, and CK5 ^±^ or CK14 ^±^); triple negative subtype (TN; HER2^−^, ER^−^, and PR^−^, HER2^−^; Figure 1).

### 2.5. cfDNA isolation from plasma samples

Cell-free DNA isolation was performed from each plasma sample. Briefly, cfDNA was extracted from ± 3 ml of plasma with the QIAamp Circulating Nucleic Acid Kit and the vacuum system QIAvac 24 Plus (Qiagen, Hilden, Germany). The Quantus fluorometer (Promega, Madison, WI, USA) and the Agilent 2200 TapeStation system (Agilent, Santa Clara, CA, USA) with the High Sensitivity D1000 ScreenTape assay were used for assessment of sample purity, cfDNA concentration and fragment size distribution according to the manufacturer's instructions. The average fragment size was determined with the Agilent 2100 Bioanalyzer Expert software, and calculated across the first three peaks 75–675 bp.

### 2.6. Design of primers and digital droplet PCR analysis for wildtype *TP53* gene and *TP53* point mutation in codon 245 in plasma samples

For the assessment of the wildtype *TP53* gene and its mutation at codon 245 in plasma samples, ddPCR assays were designed using the Bio-Rad Droplet Digital PCR Assays. Two different probes, one targeting the wildtype sequence and the other one targeting the mutation region were labeled with FAM and SUN probes to detect the wildtype (*wt*) and mutant (*mut*) *TP53* allele, respectively. These customized primers and fluorescent probes were ordered from Integrate DNA Technologies (IDT; Supplementary Table S2). In addition, synthetic positive controls with the *wt-* or *mut-TP53* sequence were designed.

One microgram (μg) of genomic DNA from plasma was digested with five units of ApoI restriction endonuclease in a total volume of 25 μl. ddPCR was performed using a QX100 Droplet Digital PCR System (Bio-rad). A reaction mixture (20 μl) containing a final concentration of 500 nM of forward and reverse primers, 2 × ddPCR Master Mix (Bio-Rad), 250 nM of FAM- and SUN-labeled probes and 80 ng DNA from each isolated plasma sample was used. Primers and probe sequences are reflected in Supplementary Table S2. Next, reaction mixtures were partitioned into an emulsion of ~20,000 droplets using QX100 Droplet Generator (Bio-Rad). Based on clearest separation of negative and positive droplet cluster with a previous standardization, thermal cycling conditions were established at 95°C for 10 min followed by 40 cycles of 95°C for 30 s and 62°C for 60 s. After thermal cycling the PCR plates were transferred to the QX100 Droplet Reader (Bio-Rad). Wild-type and mutant synthetic positive control DNA and the non-template control reactions were included in each experiment. To ensure experiment quality, wells with total droplet counts < 8,000 were considered invalid, and the experiment was repeated to obtain a sufficient number of droplets. Additionally, positive control samples carrying *wt-* and *mut-TP53* sequences were used. For each bitch, plasma was analyzed in triplicate. QuantaSoft v1.7.4.0917 (Bio-Rad Laboratories) software was used for data analysis.

### 2.7. RT-qPCR analysis from tumor tissue samples

RNA from tumor samples was isolated and quantified using a High Pure FFPET RNA isolation kit (Roche, Germany) and NanoDrop 2000 spectrophotometer (NanoDrop) respectively, following the manufacturer's instructions. An iScript™ gDNA clear cDNA synthesis kit (BioRad, Hércules, CA, USA) was used to obtain cDNA. The same probes and primers than ddPCR were used for RT-qPCR in triplicate by using the PrimeTime Gene Expression Master Mix (IDT) with a CFX96™ Real-Time PCR Detection System (Bio-Rad, Hercules, CA, USA; Supplementary Table S2). The samples were normalized to β*-actin* and relative expression was calculated by the 2^−ΔΔCt^ method to obtain the fold-change value (31).

### 2.8. Statistical analysis

In order to assess normality distribution of the data, D'Agostino and Pearson Normality test was performed. χ^2^ test or Fisher's exact test was used to examine the association between the different clinicopathological, IHC data and cfDNA parameters. When the data were not normally distributed, non-parametric tests were performed. Mann–Whitney *U*-test was used to compare differences between two groups. Data in graphs are represented as mean ± standard deviation. All *p* values ≤ 0.05 were considered statistically significant. The data were analyzed using GraphPad Prism 8 (version 3.5.0).

## 3. Results

### 3.1. Clinicopathological features of tumors

Dogs included in the study presented a single mammary tumor (*n* = 17) or multiple mammary tumors (*n* = 19). In those cases with multiple tumors, the most malignant tumor was the one considered for the data analysis. From the total, 26 tumors were diagnosed as carcinomas while 10 tumors were benign tumors. Histological subtypes of carcinomas determined that simple carcinoma was the most frequent subtype (10/26 cases, 39%) followed by mixed carcinoma (7/26 cases, 27%) and complex subtype (5/26 cases, 19%). Other special types of carcinomas such as carcinosarcomas, adenosquamous carcinomas or undifferentiated sarcomas were encompassed as “other carcinomas” representing 15% of total (4/26). According to the histological malignant grade, 12 carcinomas were classified as histological grade I (46%), five as grade II (19%) and nine as grade III (35%). On the other hand, lymphatic invasion was present in six out of 26 carcinomas (23%), tumor necrosis was present in 17 cases (65%) and 21 carcinomas (80%) showed notable tumor-associated inflammation.

Considering molecular subtypes of carcinomas, TN subtypes were the most frequent between carcinomas (9/26, 35%). Next, luminal A subtype was the second most frequent with six cases (26%), luminal B with five cases (22%) and only three cases were considered HER2^+^ subtype (13%).

### 3.2. Analysis of cfDNA concentration and correlation with clinicopathological data

**Figure 2:**
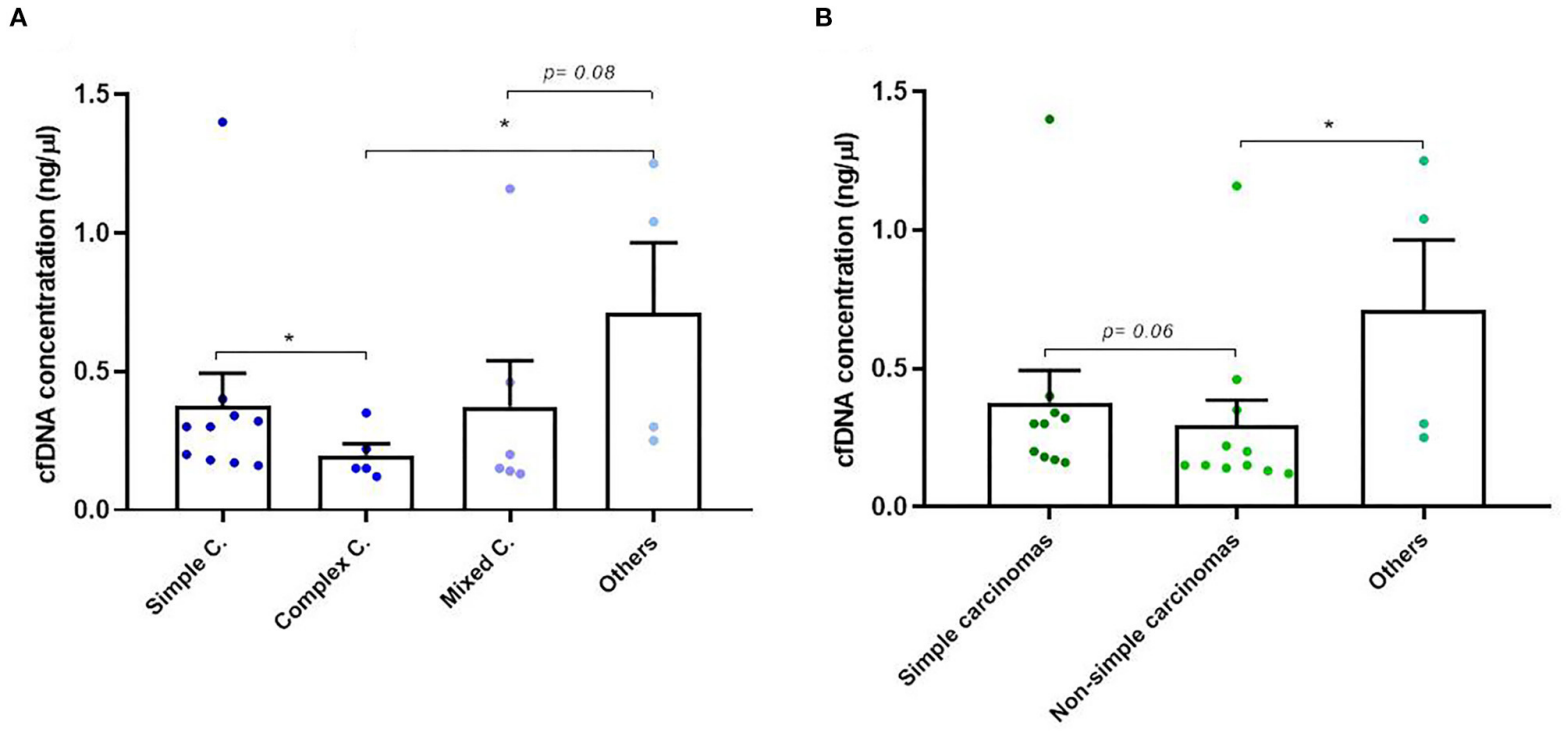
Circulating cell-free (cf) DNA concentrations in dogs with mammary carcinomas. **(A)** Scatterplot of cfDNA concentration (ng/μl) in the different histological subtypes of carcinomas **(A)** and between simple, non-simple and undifferentiated carcinomas **(B)**. ^*^p < 0.05.

**Figure 3:**
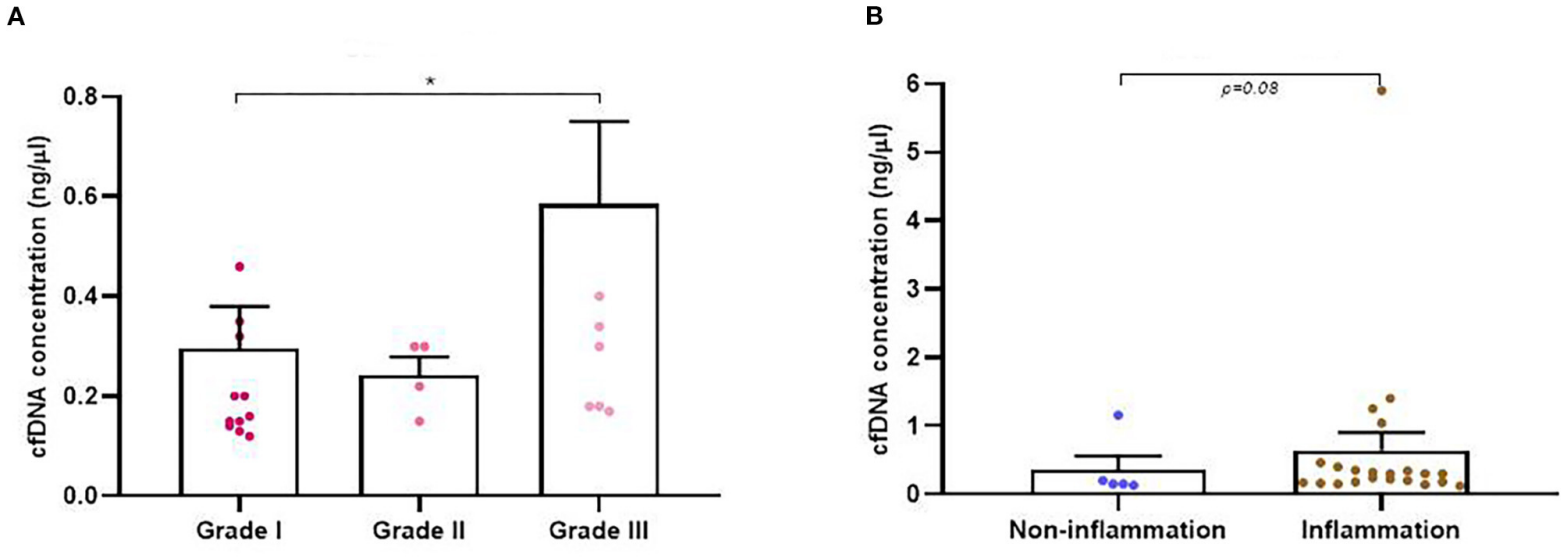
Circulating cell-free (cf) DNA concentrations in dogs with mammary carcinomas. **(A)** Scatterplot of cfDNA concentration (ng/μl) in the different malignant histological grade of carcinomas **(A)** and between carcinomas with inflammation and non-inflammation **(B)**. ^*^p < 0.05.

Isolation of plasma cfDNA from mammary tumor-bearing dogs and from healthy dogs was successfully assessed with an average of concentration of 0.91 ± 2.74 ng/μl (range 0.03–17 ng/μl). The relationship between cfDNA concentration and clinicopathological characteristics of dogs are presented in Table 1. The cfDNA concentration in tumor-bearing dogs (malignant/benign tumors) was higher (1 ng/μl) than in healthy dogs (0.25 ng/μl). Additionally, carcinomas showed higher cfDNA concentrations than benign tumors but without statistical significance (0.60 vs. 0.37 ng/μl, respectively). While no differences were observed between histological subtypes in benign tumors (Table 1) “other carcinomas” (carcinosarcomas, adenosquamous carcinomas/undifferentiated sarcomas) and simple carcinomas displayed higher cfDNA concentrations than complex carcinomas (*p* = 0.03 and *p* = 0.05, respectively) and mixed carcinomas (*p* = 0.08; Figure 2A). Interestingly, “other carcinomas” showed the highest cfDNA concentration values in comparison to non-simple carcinomas (*p* = 0.02), and cfDNA values were higher in simple carcinomas than non-simple carcinomas (*p* = 0.06; Figure 2B). On the other hand, histological grade III of carcinomas showed a significant higher cfDNA concentrations compared to grade I carcinomas (*p* = 0.03). In addition, carcinomas with tumor-associated inflammation also shown higher cfDNA concentrations than those carcinomas without inflammation (*p* = 0.08; Figures 3A, B).

**Table 1:** Association between cfDNA concentration (ng/μl) and clinicopathological features of female dogs included in the study.

**Clinical characteristics**	**Number (%)**	**cfDNA (ng/μl)**	**P-value**
Age			
< 9 years old	16 (45%)	0.41 ± 0.37	*0.31*
< 9 years old	20 (55%)	0.79 ± 0.44	
Breed			
Pure	20 (55%)	0.50 ± 0.48	*0.89*
Mixed	16 (45%)	0.63 ± 0.37	
**Tumor characteristics**			
**Number of tumors**			
Unique	17 (47%)	39.58 ± 44.43	*0.28*
Multiple	19 (53%)	39.56 ± 27.84	
**Tumor type**			
Benign	10 (27.8%)	0.37 ± 0.41	*0.45* (benign vs. malignant)
Malignant	26 (72.2%)	0.60 ± 1.14	*0.42* (malignant vs. no tumors)
No tumor	5 (12.2%)	0.25 ± 0.08	*0.48* (benign vs. no tumors)
**Histological subtype in benign tumors**			
Simple adenoma	1 (10%)	–	–
Complex adenoma	4 (40%)	0.26 ± 0.08	*0.36* (complex vs. mixed)
Mixed adenoma	3 (30%)	0.66 ± 0.70	*0.20* (mixed vs. others)
Other benign lesions	2 (20%)	0.18 ± 0.05	*0.20* (complex vs. others)
**Histological subtype in malignant tumors**			
Simple carcinoma	10 (39%)	0.38 ± 0.36	*0.05* (simple vs. complex)
Complex carcinoma	5 (19%)	0.19 ± 0.37	*0.39* (complex vs. mixed)
Mixed carcinoma	7 (27%)	0.37 ± 0.40	*0.19* (simple vs. mixed)
Other carcinomas	4 (15%)	0.71 ± 0.51	*0.18* (simple vs. others)
			*0.03* (complex vs. others)
			*0.08* (mixed vs. others)
**Histological subtype in malignant tumors**			
Simple carcinomas	10 (39%)	0.38 ± 0.36	*0.06* (simple vs. non-simples)
Non-simple carcinomas	12 (46%)	0.29 ± 0.30	*0.23* (others vs. simples)
Other malignant tumors	4 (15%)	0.69 ± 0.53	*0.05* (others vs. non-simples)
**Histological grade**			
Grade I	12 (46%)	0.29 ± 0.29	*0.03* (I vs. III)
Grade II	5 (19%)	0.24 ± 0.07	*0.12* (II vs. III)
Grade III	9 (35%)	0.58 ± 0.49	*0.33* (I vs. II)
**Lymphatic invasion**			
Yes	6 (23%)	0.67 ± 1.26	*0.44*
No	20 (77%)	0.26 ± 0.10	
**Peritumoral inflammation in malignant tumors**			
Yes	21 (80%)	0.64 ± 1.23	*0.08*
No	5 (20%)	0.35 ± 0.44	
**Necrosis in malignant tumors**			
Yes	17 (65%)	0.40 ± 0.40	*0.30*
No	9 (35%)	0.71 ± 1.38	
**Molecular subtype of carcinomas**			
Luminal A	6 (26%)	0.33 ± 0.35	*0.16* (Lum. A vs. Lum. B)
Luminal B	5 (22%)	0.48 ± 0.39	*0.12* (Lum. B vs. HER2^+^)
TN-basal	9 (35%)	0.98 ± 1.88	*0.26* (TN-basal vs. Lum. A)
			*0.20* (TN-basal vs. Lum. B)
HER2^+^	3 (13%)	0.18 ± 0.01	*0.47* (HER2^+^ vs. Lum. A)
			*0.22* (HER2^+^ vs. TN-basal)
No determined	3 (4%)	0.61 ± 0.56	*0.13* (no det vs. Lum. A)
			*0.39* (no det vs. Lum. B)
			*0.15* (no det vs. HER2^+^)
			*0.28* (no det vs. TN-basal)

Italic numbers are the *p*-values.

Regarding molecular subtypes of carcinomas, TN subtype shown the highest cfDNA concentrations in plasma (0.98 ng/μl) but no significant differences were observed with respect to the other molecular subtypes (Table 1). Finally, gender, age, breed, the lymphatic invasion, number of tumors or presence of necrosis were not linked to plasma cfDNA concentration (Table 1).

### 3.3. Analysis of cfDNA fragmentation and correlation to clinicopathological data

**Figure 4:**
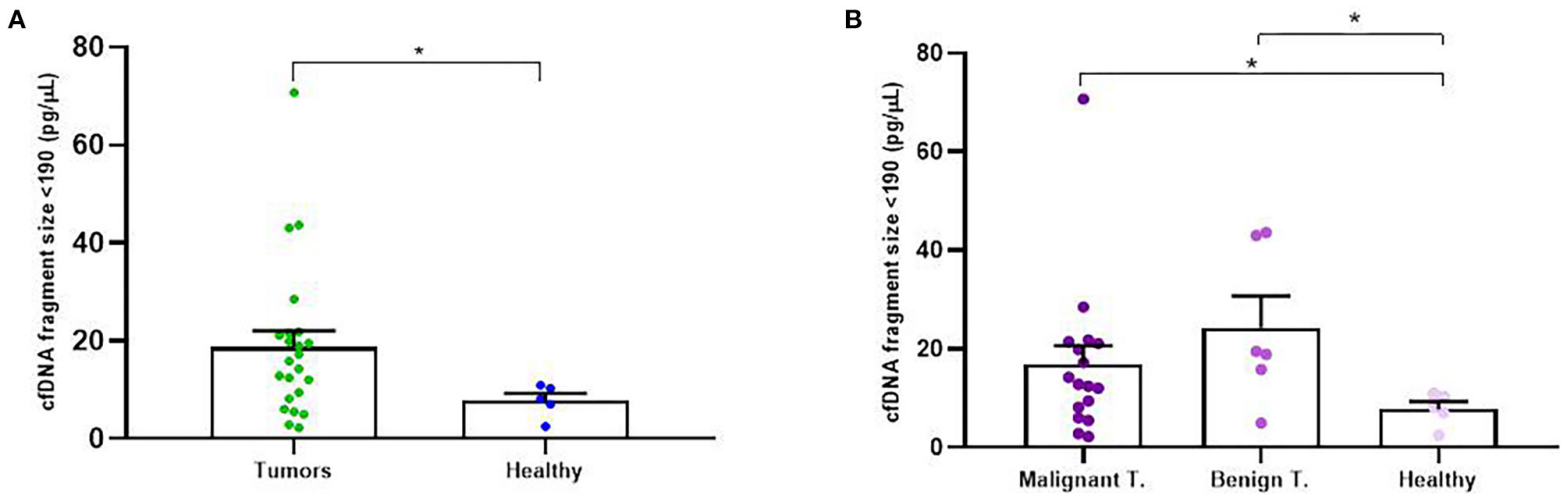
Circulating cell-free (cf) DNA fragmentation size (<190 bp, pg/μl) in dogs with mammary carcinomas. Scatterplot of cfDNA fragmentation size <190 bp (pg/μl) in dogs with tumors and healthy dogs **(A)** and between malignant and benign tumors and controls dogs **(B)**. ^*^p < 0.05.

**Figure 5:**
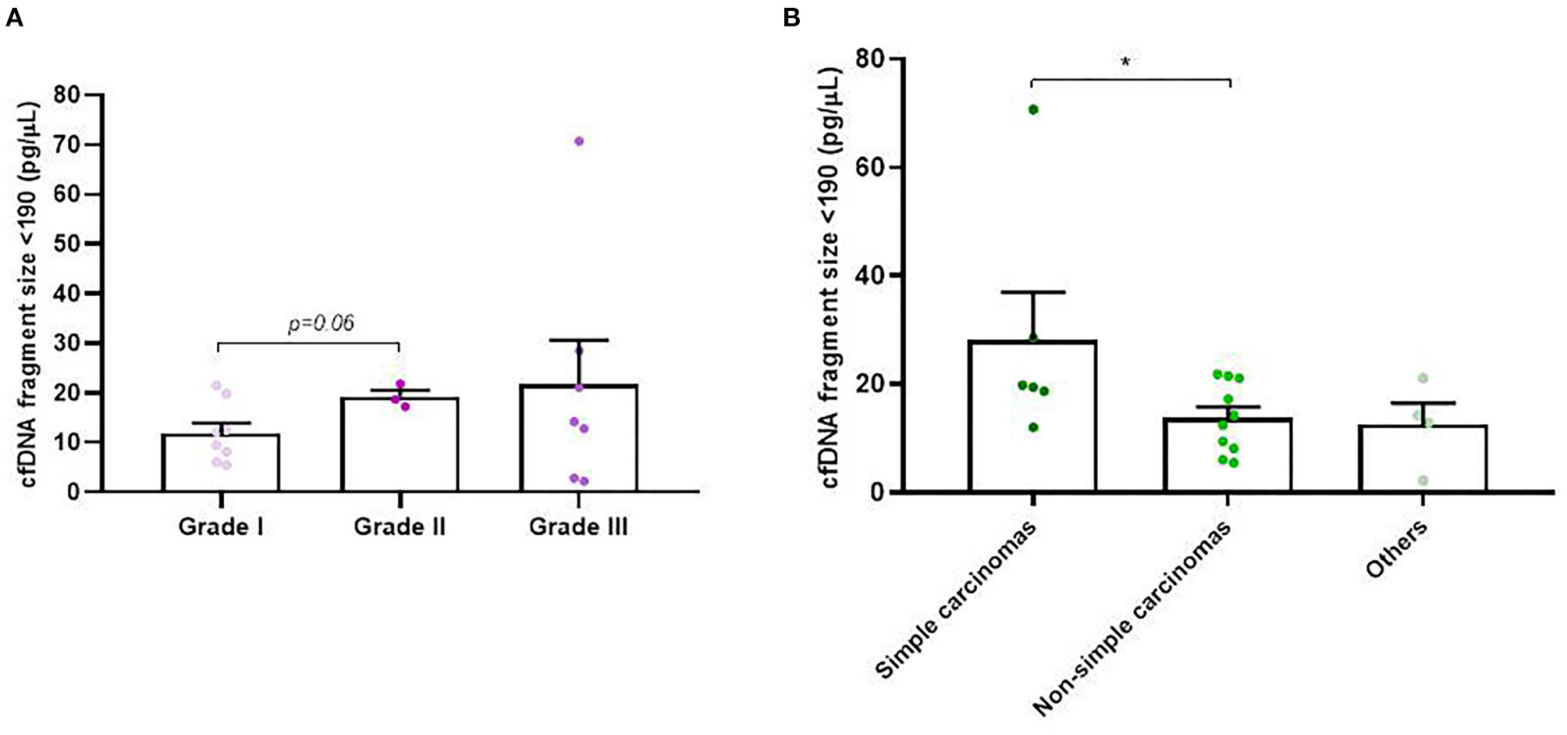
Circulating cell-free (cf) DNA fragmentation size (<190 bp, pg/μl) in dogs with mammary carcinomas. Scatterplot of cfDNA fragmentation size <190 bp (pg/μl) between different histological grade **(A)** and between simple, non-simple carcinomas and undifferentiated carcinomas **(B)**. ^*^p < 0.05.

In addition to concentration, cfDNA fragment size was also analyzed in order to evaluate its association with the rest of the clinicopathological parameters. Firstly, we decided to focus on cfDNA fragmentation less than 190 bp (short cfDNA fragments) which is known to be specifically enriched for DNA fragments derived from tumors (20). Thus, in our study it was noted that tumor-bearing dogs had significantly higher concentration of short-fragments than healthy control dogs (*p* = 0.02; Figure 4A). Likewise, malignant and benign-bearing dogs showed a higher concentration of cfDNA short-fragments than healthy dogs (*p* = 0.05 and *p* = 0.02, respectively, Figure 4B). Moreover, grade II and III carcinomas also trended to show higher rates of short-fragments than grade I carcinomas (*p* = 0.06; Figure 5A). In addition, when cfDNA fragmentation was analyzed and correlated with the histological subtype, simple carcinomas displayed significantly higher cfDNA concentrations of short-fragments than non-simple carcinomas (Figure 5B).

Regarding the rest of parameters and the ranges of fragments analyzed (< 400 bp, 450–650 bp, 650–850, and 1.000–1,200 bp), no statistically significant differences were observed between them.

### 3.4. Quantitative detection of wt-*TP53* expression and *TP53*-point mutation detection in cfDNA

**Figure 6:**
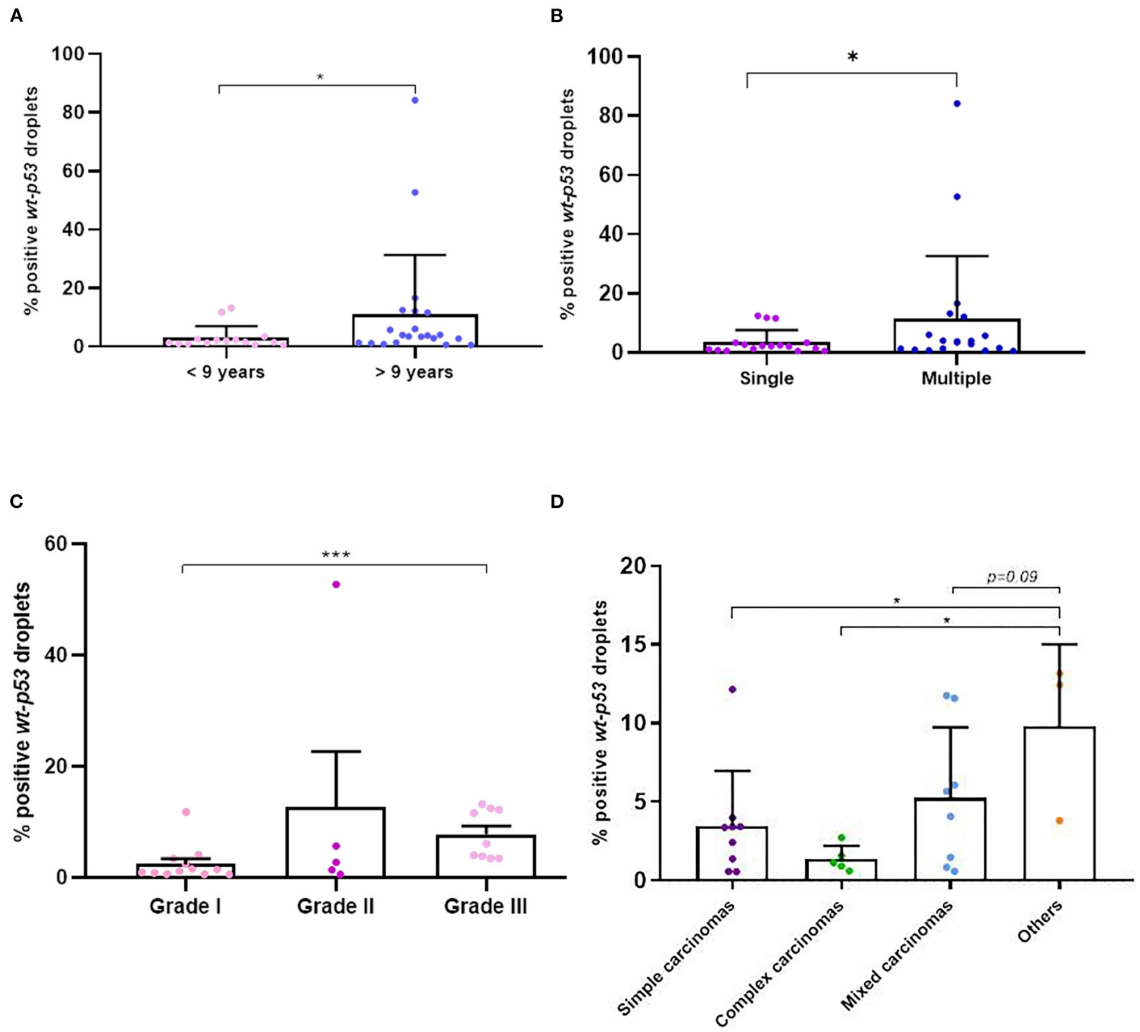
Percentage of positive *wt-p53* droplets detected in cfDNA in dogs with mammary carcinomas. Scatterplot of percentage of positive *wt-p53* droplets in dogs with <9 years old and >9 years old **(A)**, between dogs with single and multiple tumors **(B)**, between different malignant histological grades **(C)** and the different histological subtypes of carcinomas **(D)**. ^*^p < 0.05 and ^***^p < 0.001.

We were successful to amplified *wt-TP53* gene in plasma cfDNA in all cases but no positive samples for *TP53*-point mutation at codon 245 (*mut-TP53 gene*) could be confirmed. Interestingly, the *wt-TP53* expression analysis by ddPCR showed significant association with some of the clinicopathological data of the dogs (Table 2). Thus, the percentage of positive *wt-TP53 (wt-TP53*^+^) droplets by ddPCR was higher in older dogs (*p* = 0.02), with multiple tumors (*p* = 0.06) and with histological grade III (*p* = 0.0005) than in their countersparts (Figures 6A–C). In addition, dogs with “other carcinomas” had an even greater *wt-TP53* expression than those that had simple (*p* = 0.03) complex (*p* = 0.03) or mixed (*p* = 0.09) carcinomas (Figure 6D). Regarding molecular subtype of carcinomas, we also appreciated that bitches with TN subtype were the one with highest concentration of *wt-TP53*^+^ droplets than the rest of molecular subtypes but without statistically significance (Table 2).

**Table 2:** Association between percentage of droplet *wt-TP53* expression analysis determined by ddPCR and clinicopathological features of female dogs included in the study.

**Clinical characteristics**	**Number (%)**	**% droplet *p53*wt**	**P-value**
Age			
< 9 years old	16 (45%)	3.05 ± 3.90	*0.02*
< 9 years old	20 (55%)	11.02 ± 2.24	
Breed			
Pure	20 (55%)	8.93 ± 18.42	*0.65*
Mixed	16 (45%)	6.48 ± 4.12	
**Tumor characteristics**			
**Number of tumors**			
Unique	17 (47%)	3.56 ± 4.09	*0.06*
Multiple	19 (53%)	11.36 ± 21.29	
Tumor type			
Benign	10 (27.8%)	3.32 ± 5.03	*0.23* (benign vs. malignant)
Malignant	26 (72.2%)	6.25 ± 10.37	*0.17* (malignant vs. no tumors)
No tumor	5 (12.2%)	1.35 ± 0.58	*0.33* (benign vs. no tumors)
**Histological subtype in benign tumors**			
Simple adenoma	1 (10%)	–	–
Complex adenoma	4 (40%)	1.78 ± 0.91	*0.40* (complex vs. mixed)
Mixed adenoma	3 (30%)	6.96 ± 8.34	*0.11* (mixed vs. others)
Other benign lesions	2 (20%)	0.18 ± 0.05	*0.11* (complex vs. others)
**Histological subtype in malignant tumors**			
Simple carcinomas	10 (39%)	2.47 ± 3.46	0.97 (simple vs. non-simples)
Non-simple carcinomas	12 (46%)	6.48 ± 13.12	0.63 (others vs. simples)
Other carcinomas	4 (15%)	6.67 ± 6.63	0.54 (others vs. non-simples)
**Histological subtype in malignant tumors**			
Simple carcinoma	10 (39%)	3.48 ± 3.50	*0.11* (simple vs. complex)
Complex carcinoma	5 (19%)	1.39 ± 0.82	*0.11* (complex vs. mixed)
Mixed carcinoma	7 (27%)	5.26 ± 4.47	*0.16* (simple vs. mixed)
Other carcinomas	4 (15%)	9.81 ± 5.20	*0.03* (simple vs. others)
			*0.03* (complex vs. others)
			*0.09* (mixed vs. others)
**Histological grade**			
Grade I	12 (46%)	2.44 ± 3.14	*0.0005* (I vs. III)
Grade II	5 (19%)	12.62 ± 22.51	*0.11* (II vs. III)
Grade III	9 (35%)	7.78 ± 4.41	*0.19* (I vs. II)
**Lymphatic invasion**			
Yes	6 (23%)	6.67 ± 11.67	0.30
No	20 (77%)	4.84 ± 4.11	
**Peritumoral inflammation in malignant tumors**			
Yes	21 (80%)	4.08 ± 4.17	*0.44*
No	5 (20%)	6.90 ± 11.62	
**Necrosis in malignant tumors**			
Yes	17 (65%)	4.28 ± 4.47	*0.19*
No	9 (35%)	7.34 ± 12.41	
**Molecular subtype of carcinomas**			
Luminal A	6 (26%)	2.97 ± 4.35	*0.12* (Lum. A vs. Lum. B)
Luminal B	5 (22%)	4.19 ± 4.47	*0.99* (Lum. B vs. HER2^+^)
TN-basal	9 (35%)	9.48 ± 16.60	*0.07* (TN-basal vs. Lum. A)
			*0.30* (TN-basal vs. Lum. B)
HER2^+^	3 (13%)	2.96 ± 1.32	*0.13* (HER2^+^ vs. Lum. A)
			*0.43* (HER2^+^ vs. TN-basal)
No determined	3 (4%)	0.61 ± 0.56	*0.13* (no det vs. Lum. A)
			*0.39* (no det vs. Lum. B)
			*0.15* (no det vs. HER2^+^)
			*0.28* (no det vs. TN-basal)

Italic numbers are the *p*-values.

### 3.5. Quantitative detection of *wt-TP53* expression and *TP53*-point mutation detection in tumor tissue

**Figure 7:**
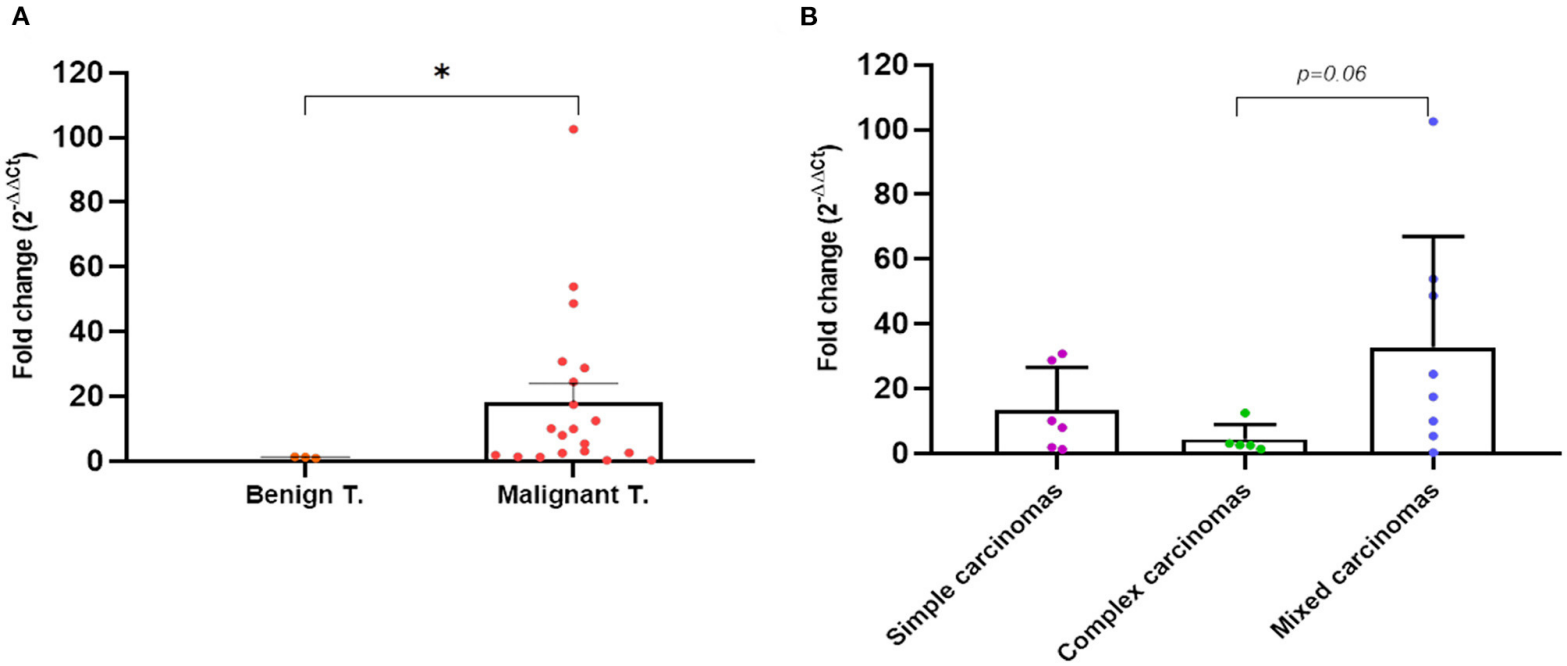
Quantitative PCR fold-change *p53* expression differences in tumor tissues of dogs with mammary carcinomas. Scatterplot of PCR fold-change expression between benign and malignant tumors **(A)** and between simple, complex, and mixed carcinomas **(B)**. ^*^p < 0.05.

In parallel to the analysis in plasma, in 26 dogs (four with benign and 22 with mammary carcinomas), the expression of the *wt-TP53* gene and *mut-TP53* gene was carried out by RT-qPCR from tumor tissue. The mean concentration of RNA extracted from the tumor tissue samples was 261.84 ng/μl (range 32.96 ng/μl−621.15 ng/μl). As observed in plasma, the mutation under study was not detected in any of the tumor tissues analyzed, but there was differential expression regarding to *wt-TP53* gene expression. The fold-change analysis with respect to the analyzed benign tumors, determined that the carcinomas showed an average of 18 times more (18 fold-changes) expression of the *wt-TP53* gene than the *wt-TP53* gene in benign tumors (*p* = 0.04, Figure 7A). As observed in plasma, complex carcinomas were the tumors with a lower fold-change compared to benign tumors and compared to simple and mixed carcinomas (*p* = 0.06, Figure 7B). Regarding molecular subtypes, no significant differences were observed.

### 3.6. Correlation of *wt-TP53* gene expression in plasma and tumor tissue

**Figure 8:**
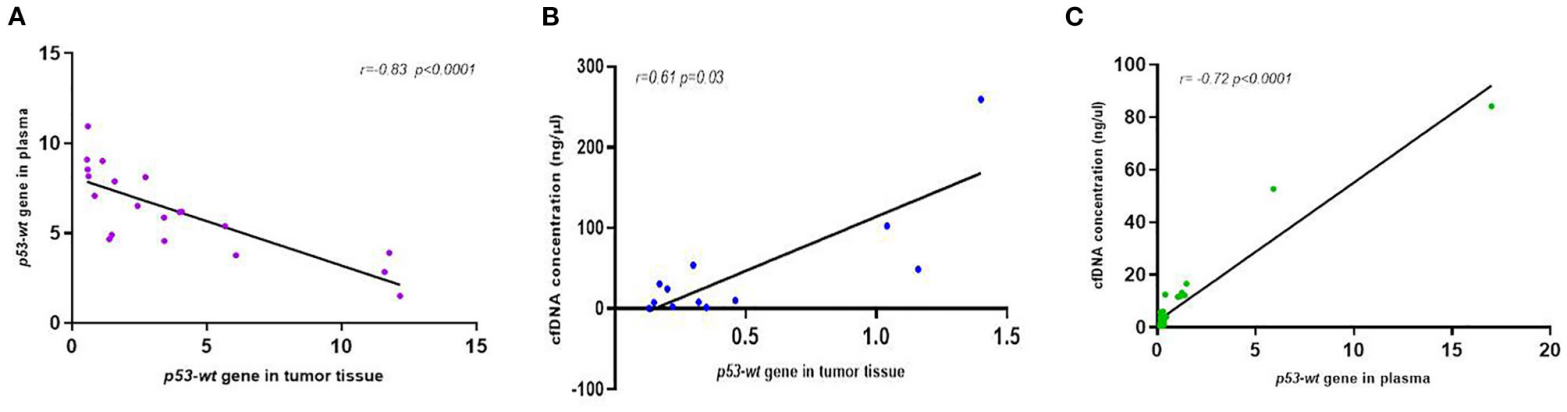
Correlation analysis between *p53*-expression in plasma and tumor tissue **(A)** and between cfDNA concentration and *p53*-expression in tumor tissue **(B)** and plasma **(C)** of dogs with mammary carcinomas.

The comparative analysis of *wt-TP53* gene expression at the plasma and tissue level tumor determined a high correlation between both (*r* = −0.83*, p* < 0.0001; Figure 8A), so that those animals that presented higher values of expression of the gene *wt-TP53* (% *TP53-wt* droplets^+^) at the plasma level showed lower Ct values in tissue by RT-qPCR. In the same way, the expression of *wt-TP53* in tissue and plasma also showed a significant correlation with the total concentration of cfDNA in plasma (*r* = 0.61, *p* = 0.03 and *r* = 0.72 *p* < 0.0001, respectively), observing higher levels of *wt-TP53* in those cases with higher concentrations of cfDNA (Figures 8B, C).

## 4. Discussion

The detection of cfDNA by liquid biopsy is considered one of the most advanced and minimally invasive tools that have emerged in the recent years in the field of human oncology and, more recently, in veterinary medicine (32–34). In the last years, an increasing number of studies demonstrate the relevance of cfDNA as an attractive tumor biomarker with great potential for the amount of information it provides about the tumor progression and prognosis as well as genetic alterations of certain cancer-related genes (*BRAF, EGFR, TP53, KRAS, PIK3CA…*), in a non-invasive way (1, 3, 4).

One of the outstanding results from this study is the analysis of cfDNA concentration and fragmentation as well as the *TP53* gene expression in the plasma of dogs with mammary tumors and its association with clinicopathological data. Thus, as early studies in human medicine, we found that plasma cfDNA concentrations were elevated in tumor-bearing dogs compared with healthy dogs and with poor prognosis characteristics: simple or undifferentiated carcinomas, higher histological grade of tumors and presence of peritumoral inflammation (4–6). While several studies in human medicine have observed the usefulness of cfDNA concentration as an early predictor of prognosis, aggressiveness or metastasis, there has been a lack of studies in veterinary oncology (10, 12, 32, 33). In the present study, the concentration of cfDNA in dogs with benign tumors was higher than that in healthy dogs, but lower than that in carcinoma-bearing dogs as previously has been reported for humans (35–38). As we have observed here, some studies in CMTs and lymphomas, have found a correlation between ctDNA concentration and worse prognosis and severity of the tumor (10, 12, 33). In another study, the number of repetitive DNA sequences (CAN SINE sequences) was evaluated in cfDNA of dogs with mammary tumors and observed a direct association with a worse prognosis (39). However, it should be noted in this study, that cfDNA concentration in carcinomas does not vary significantly depending on molecular subtype, lymphatic invasion or presence of necrosis.

Another interesting finding was the elevated percentage of short-fragments in the ctDNA of animals with malignant and benign tumors compared to healthy dogs. Interestingly, grade II and III carcinomas showed higher concentration of cfDNA short-fragments than grade I and simple carcinomas displayed higher cfDNA short-fragments than non-simple carcinomas. According to our results, Beffagna et al. (12), quantified the amount of short and long cfDNA fragments by qPCR, and demonstrated higher cfDNA short-fragments than healthy dogs or non-neoplastic diseased dogs. Coinciding with previous publications in human medicine (12) we have been able to quantify and analyse cfDNA fragments by using a more specific fragment analyzer system for cancer patients. The fragment length of cfDNA tends to be shorter (< 190 bp) in more malignant tumors (21, 40, 41). It has also been suggested that higher ctDNA fragmentation (and therefore a greater number of short-fragments) may be present in cancer cell apoptosis compared to non-cancer cell apoptosis. This would imply that the degree of fragmentation during apoptosis may be different between tumor-bearing and healthy individuals (42). Likewise, the canine cfDNA fragmentome is poorly understood at this time, requiring future studies, with higher number of dogs, to this regards to fully understand its potential as a biomarker for clinical use.

Accordingly, the determination of ctDNA levels in plasma, as well as the analysis of the size of the ctDNA fragments, can be detected using a non-invasive technique, providing significant clinical relevance in veterinary oncology and contribute to the better definition and detection of tumors.

Finally, it is worth highlighting the findings regarding the basal expression of the *TP53* gene in plasma of dogs with mammary tumors. In both human and animal patients, *TP53* overexpression, primarily assessed by IHC techniques in tumor tissues, is linked to poor prognosis and tumor progression confirming the usefulness of this oncogene as a tumor biomarker (43–46). In our study, a differential expression of the *TP53-wt* gene has been observed in plasma and in tumor tissue in dogs.

From our knowledge, this is the first study that analyzes the expression of this tumor suppressor gene using the ddPCR technique in CMTs. Thus, we observed that the percentage of expression of *TP53-wt* is higher in dogs with tumors than in healthy dogs and in the case of “other carcinomas” the expression is increased with respect to simple or complex carcinomas. As in human breast cancer, *TP53* gene is one of the most frequently mutated gene in CMTs (15%−20%) (43). In addition, in breast carcinoma, overexpression of *TP53* is associated with point mutations within highly conserved regions of in exon 5–8. To date, the most frequent *TP53* mutations that has been describe in dogs are located in exon 7 at codons 236, 245 and 249 (47–49) but no data from canine plasma samples have been previously described. The most likely reason why the point mutation at codon 245 has not been detected in our study could be: that the analyzed samples do not carry said mutation, or that it was at a lower frequency than ddPCR detection limit. Therefore, in order to confirm these results, it is essential that other studies are carried out including a larger number of animals as well as the analysis of other potential key point mutations for this cancer related-gene. Taking into account that human and canine *TP53* gene shows 86.3% homology, makes the dog a good model for comparative studies of this oncogene in human oncology.

Interestingly, it was also observed that the expression of the *TP53-wt* gene in plasma showed a high concordance with the levels of expression of the gene found in tumor tissue. Moreover, a correlation was also observed between a higher expression of the *TP53-wt* gene and the presence of a higher amount of cfDNA in plasma, which would also determine a poor prognosis indicator. These findings are quite novel since this is the first study that analyses this oncogene at the plasma and tissue level at the same time and determine that the *TP53* expression in plasma faithfully reflects what is observed in the tumor.

In summary, the results obtained from this work indicate, as occurs in humans, that higher cfDNA concentration, short-fragments and higher *TP53* expression (in plasma and tissue) in CMTs correlates with worse prognosis clinicopathological parameters. The validation and standardization of this methodology in CMTs will offer a unique, safe and easy opportunity for the diagnosis, prognosis and treatment of animals with mammary tumors. Future research in the field will confirm the prognostic and predictive value of ctDNA and its fragments, as well as the analysis of target mutations in veterinary oncology and make this methodology available to clinical veterinary centers.

## Data availability statement

The original contributions presented in the study are included in the article/Supplementary material, further inquiries can be directed to the corresponding author.

## Ethics statement

The animal study was reviewed and approved by Comité de Bioética y Seguridad and Universidad de Córdoba. Written informed consent was obtained from the owners for the participation of their animals in this study.

## Author contributions

SG-L, RS-C, and YM designed this study and critically revised the manuscript. MDF performed IHQ analysis and interpretation of data. AR performed the gene analysis of plasma and tissue samples. JAF and AR-A contributed to the collection, clinical characterization of clinical samples, and elaboration of the manuscript. All authors read and approved the final manuscript.
