# Supplementary material for: Analysis of cell-free DNA concentration, fragmentation patterns and TP53 gene expression in mammary tumor-bearing dogs: A pilot study
Source: Front Vet Sci. 2023 Mar 29;10:1157878. doi: 10.3389/fvets.2023.1157878 (PMC10090457; doi:10.3389/fvets.2023.1157878)
Supplement: Supplementary file 1 [file Table_1.docx]

**Table S1.** List of primary antibodies used for immunohistochemistry analysis.

| **Primary Antibody** | **Host primary antibody** | **Dilution** | **Antigen retrieval** | **Clone** | **Source** |
| --- | --- | --- | --- | --- | --- |
| PR | Mouse monoclonal | 1:500 | Citrate buffer pH6 | 10 A9 | Immunotech |
| ER | Mouse monoclonal | 1:200 | Citrate buffer pH6 | 6F11 | Zymed Laboratories |
| c-erbB-2 | Mouse monoclonal | 1:100 | Citrate buffer pH6 | A0485 | Dako |
| Ki-67 | Mouse monoclonal | 1:75 | Citrate buffer pH9 | MIB -1 | Dako, Madrid, Spain |
| CK14 | Mouse monoclonal | 1:40 | Citrate buffer Ph6 | LL002 | BioGenex |
| CK5 | Mouse monoclonal | 1:150 | Citrate buffer pH6 | V9 | Novocastra |
