# Supplementary material for: Analysis of cell-free DNA concentration, fragmentation patterns and TP53 gene expression in mammary tumor-bearing dogs: A pilot study
Source: Front Vet Sci. 2023 Mar 29;10:1157878. doi: 10.3389/fvets.2023.1157878 (PMC10090457; doi:10.3389/fvets.2023.1157878)
Supplement: Supplementary file 2 [file Table_2.docx]

**Table S2.** List of primers, probes and sequences used in the present study.

| **Gen name** | **Primer** | **Sequence (5´-3´)** |
| --- | --- | --- |
| p53- codon 245 | Forward | CTCAGGTTGGCTCTGACTATAC |
|  | Reverse | GAGTCTTCCAGGGTGATGATAG |
| β-actin | Forward | CCGCCTATTCCAGGATTCTCT |
|  | Reverse | GGACCTTCCCAACCCTGTTAG |
| **Name** | **Probe** | **Sequence** |
| Codon245_C_Allele/ mutated | FAM | /56-FAM/TT+CAT+G+G+CT+CC/3IABkFQ/ |
| Codon245_G_Allele/wildtype | SUN | /5SUN/TT+CAT+G+C+CT+CC/3IABkFQ/ |
| **Name** | **Exon** | **Sequence (5´-3´)** |
| Canis lupus familiaris | **7** | ACCCTGGGCCTACCTTCTACCTCAGGTTGGCTCTGACTATACCACCATCCACTACAACTACATGTGTAACAGTTCCTGCATGGGAG**G**/**C**CATGAACCGGCGGCCCATCCTCACTATCATCACCCTGGAAGACTCCAGGTAGGGACCTGCCTGCCACCCT |
